# TAT Nanobody Exerts Antiviral Effect against PRRSV In Vitro by Targeting Viral Nucleocapsid Protein

**DOI:** 10.3390/ijms24031905

**Published:** 2023-01-18

**Authors:** Jiahui Ren, Hong Duan, Haoxin Dong, Shuya Wu, Yongkun Du, Gaiping Zhang, Angke Zhang

**Affiliations:** 1 College of Veterinary Medicine, Henan Agricultural University, Zhengzhou 450046, China; 2 International Joint Research Center of National Animal Immunology, College of Veterinary Medicine, Henan Agricultural University, Zhengzhou 450046, China

**Keywords:** PRRSV, nanobody, nucleocapsid protein, trans-activator transcription, antiviral effect

## Abstract

Porcine reproductive and respiratory syndrome (PRRS) is caused by the PRRS virus (PRRSV), which has brought huge economic losses to the pork industry worldwide since its first discovery in the late 1980s in North America. To date, there are no effective commercial vaccines or therapeutic drugs available for controlling the spread of PRRSV. Due to their unique advantages of high affinity and high specificity, nanobodies (Nbs) have received increasing attention in the process of disease diagnosis and treatment. Trans-activator transcription (TAT) can serve as a vector to carry specific proteins into cells by passing through cell membranes. In our previous study, a specific Nb against the PRRSV nucleocapsid (N) protein was screened using phage display technology. For this study, we developed a novel recombinant protein constituting a TAT-conjugated Nb, which we call TAT-Nb1. The target cell entry efficiency of TAT-Nb1 and its effect on PRRSV infection and replication were then investigated. Our results indicate that TAT delivered Nb1 into Marc-145 cells and porcine alveolar macrophages (PAMs) in a dose- and time-dependent manner. Furthermore, TAT-Nb1 dose-dependently suppressed PRRSV infection and replication, where this antiviral effect was independent of PRRSV strain. Co-immunoprecipitation results revealed that Nb1 efficiently interacted with the N protein of PRRSV. Taken together, the presented results suggest that TAT-Nb1 can effectively suppress PRRSV replication, and it may be considered as a new anti-PRRSV candidate drug.

## 1. Introduction

Porcine reproductive and respiratory syndrome (PRRS) is characterized by reproductive disorders in female pigs and respiratory distress in pigs of all ages, and it has led to enormous economic losses in the swine industry of major pig-producing countries worldwide [1]. At present, PRRS remains a major concern for the pig industry, due to the continuous emergence of recombinant mutant PRRSV strains, the poor clinical protection of commercial vaccines, and the lack of effective antiviral drugs and treatment strategies [2,3,4].

PRRS virus (PRRSV), the pathogen that causes PRRS, is an enveloped, single-stranded, positive-sense RNA virus, belonging to the *Nestoviridae*, *Arterioviridae*, and *Arterioviridae* families [5]. The PRRSV genome is about 14.9–15.5 kb and contains at least 10 open reading frames (ORFs) [6]. PRRSV strains are currently divided into two genotypes: genotype I, or European-like isolates (Lelystad Virus) [7], and genotype II, or North American-like isolates [8]. The nucleocapsid (N) protein of PRRSV is the most conserved protein in different strains, which is encoded by the viral ORF7 gene [9] and is the most abundant viral protein that can be detected after infecting host cells. The PRRSV N protein participates in viral assembly and assembles with viral RNA to form the virus nucleocapsid, simultaneously conferring high immunogenicity and reactivity, making it an ideal target for the design of antiviral drugs against PRRSV infection [10,11]. Porcine alveolar macrophages (PAMs) are the main target cells for PRRSV replication and proliferation after the infection of live pigs by the virus [12]. At the same time, another PRRSV-susceptible cell line, African green monkey kidney cell (Marc-145), is the most widely used cell model for studying PRRSV infection and replication characteristics in vitro [13]. These two cells provide effective tools for in vitro screening of anti-PRRSV drugs.

A distinct type of antibody fragment, termed VHH or nanobody (Nb), is derived from heavy-chain-only antibodies that circulate in the serum of camelids. Compared with traditional monoclonal antibodies, nanobodies (Nbs) have many unique characteristics, such as small protein molecular weight, easy genetic manipulation, high specificity and high affinity for antigens, and so on [14,15]. Due to small protein size and unique spatial conformations, Nbs can recognize protein cavities or epitopes inaccessible to conventional antibodies [16]. Some studies have shown that Nbs targeting human host viruses (e.g., HIV and SARS-CoV-2) or animal host viruses (e.g., PRRSV) can obviously restrain the replication and proliferation of these viruses in host cells, showing good antiviral activity [17,18]. However, due to the selective permeability of the cell membrane, biomacromolecules such as Nbs cannot freely pass through the cell membrane, thus severely limiting their application as novel antiviral drugs. Therefore, developing an efficient, safe, and non-toxic Nb delivery system to deliver Nbs into cells to exert biological functions such as antiviral activity can effectively promote the development and clinical application of Nbs, which can also provide a pathway for the development of new anti-PRRSV drugs.

Cell-penetrating peptides (CPPs), also known as protein transduction domains (PTDs), are short peptides of 5–30 amino acids that have the ability to pass through tissues and cell membranes without interacting with specific receptors [19]. Some reported CPPs, such as trans-activator transcription (TAT), VP22 (derived from HSV-1), penetratin, and so on, can be used as carriers to deliver specific proteins, small molecules, nucleotides, or other peptides into cells through the cell membrane without causing any cytotoxicity [20,21,22]. Of these CPPs, an 11-amino-acid TAT peptide including ^47^YGRKKRRQRRR^57^ residues, which was the first reported CPP encoded by human immunodeficiency virus type 1 (HIV-1), is effective in mediating the transmembrane transduction of various kinds of proteins [23,24]. For instance, TAT-mediated delivery of HIV-1 Nef effectively improved the immune response in mice [25]; furthermore, a TAT-fused Nb against survivin effectively passed into HepG2 cells, inhibiting target cell proliferation, promoting cell apoptosis, and exerting anti-cancer activity [26].

In our previous research, one specific Nb against the PRRSV N protein, Nb1, has been screened through the immunization of a Bactrian camel and phage display technology [27]. Based on this former study, TAT peptides fused with the specific Nb1 against PRRSV N protein and an irrelevant nanobody Nb53 were prepared, and the delivery effects of the TAT-Nb1 and -Nb53 protein into Marc-145 cells or PAMs were determined. Then, the effect of TAT-Nb1 on PRRSV replication was evaluated in vitro. Our results indicated that TAT-Nb1 and -Nb53 were successfully transduced into both Marc-145 cells and PAMs, and no marked cytotoxicity was observed within the range of concentration used. Notably, TAT-Nb1 but not -Nb53 obviously suppressed PRRSV replication in vitro. Our current findings suggest that TAT-Nb1 may have potential as a therapeutic agent in the treatment of PRRSV infection.

## 2. Results

### 2.1. Expression and Purification of the TAT-Nbs Proteins

**Figure 1:**
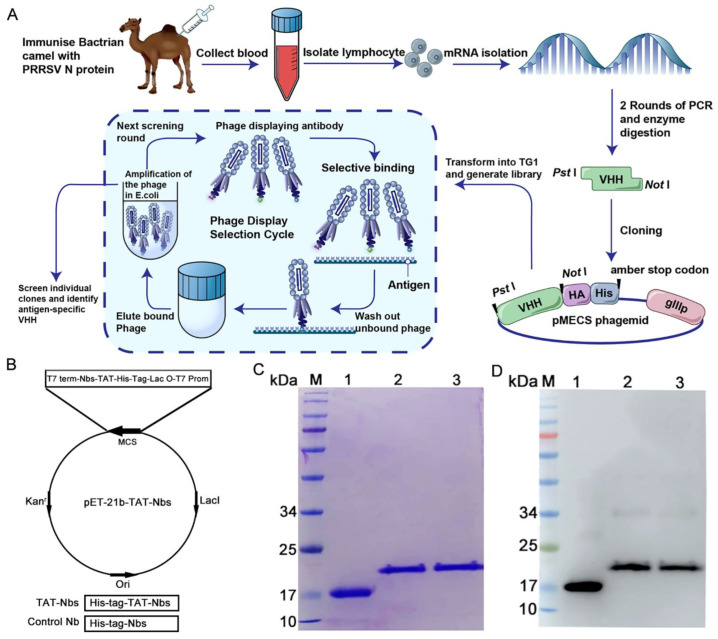
Expression and purification of the recombinant TAT-Nb1, -Nb53, and Nb1. (**A**) Schematic diagram of antigen-specific nanobody screening. (**B**) Construction of the TAT-Nbs expression system using pET-21b vector. Diagram of the construction of vector expressing Nb1 and TAT-Nbs proteins. Expression of Nbs and Nb1 in *E.coli* BL21 were induced by IPTG. After purification using a Ni-column, the purified proteins were subjected to SDS-PAGE analysis (**C**) and Western blot detection (**D**), respectively. M: molecular weight markers; Lane 1: TAT protein control; Lane 2: purified TAT-Nb1 protein; Lane3: purified TAT-Nb53 protein.

The screening of specific nanobodies against the PRRSV N protein has been described in our previous work [27], and the screening process is depicted in Figure 1A. The TAT-Nb1 and -Nb53 protein expression vectors were constructed by cloning the cDNA of TAT-Nb1 or -Nb53 into a pET-21b plasmid, respectively. The constructed recombinant vector included a continuous cDNA encoding Nb1 or Nb53, a TAT peptide, and an amino-terminal tag of six histidines (Figure 1B). At the same time, a vector expressing only Nb1 was also constructed as a control. A Ni-column was used to purify TAT-Nb1 or -Nb53 and control the Nb1 protein. After dialysis with gradient urea, the above proteins were finally dialyzed into PBS. The purity of the TAT-Nb1 or -Nb53 protein and control Nb1 protein were assessed by SDS-PAGE and Western blot analysis. The SDS-PAGE results demonstrated that His-tagged TAT-Nb1, -Nb53, and Nb1 proteins were successfully purified, and high-purity target proteins were obtained with the expected size of 15/19 kDa (Figure 1C). Furthermore, the Western blotting results suggested that the TAT-Nb1, -Nb53, and Nb1 proteins could react with anti-His mAb (Figure 1D). The concentrations of the purified TAT-Nbs were determined, aliquoted, and stored at −80 ℃ until further experiments.

### 2.2. Transduction of TAT-Nbs Protein into Marc-145 Cells or PAMs

**Figure 2:**
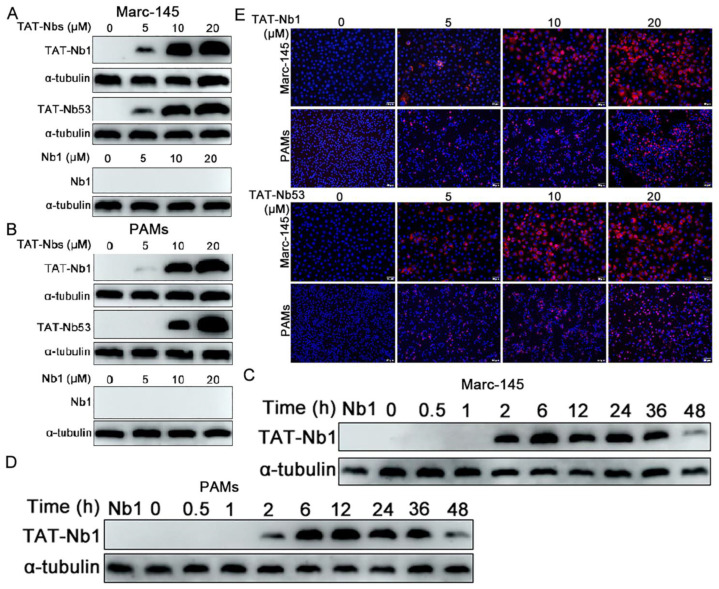
Transduction of TAT-Nb1 and -Nb53 proteins into Marc-145 cells and PAMs. Marc-145 cells (0.5 × 10^5^ cell/well) and PAMs (0.5 × 10^6^ cell/well) were seeded into 24-well cell culture plates. When reaching about 80% confluence, the cells were incubated with TAT-Nb1, -Nb53 (0, 5, 10, and 20 μM), and control Nb1 protein for 6 h. Then, Western blot was performed to analyze the entry of TAT-Nbs or Nb1 into Marc-145 cells (**A**) and PAMs (**B**). Then, 20 μM TAT-Nb1 or -Nb53 was added to Marc-145 cells (**C**) and PAMs (**D**), respectively, to analyze the stability of transduced TAT-Nbs. (**E**) IFA detection of the transduced TAT-Nbs protein into Marc-145 cells (**E**) and PAMs (**F**). Scale bar = 50 μm.

As TAT is a transmembrane peptide, the capacity of TAT-Nb1 or -Nb53 to enter target cells was first determined. Marc-145 cells or PAMs were inoculated with 0, 5, 10, or 20 μM of TAT-Nb1 or -Nb53 for 6 h, after which the protein levels of TAT-Nb1 or -Nb53 were detected by Western blotting. Both TAT-Nb1 and -Nb53 protein were transduced into the target cells in a dose-dependent manner within the range of doses used (Figure 2A,B). Next, the stability of transduced TAT-Nb1 or -Nb53 protein in both Marc-145 cells and PAMs was assessed. Both cells were treated with 20 μM TAT-Nb1 or -Nb53 protein for different time periods (0–48 h), and the amount of protein entering target cells was determined by Western blotting. As indicated in Figure 2C,D, the transduction of TAT-Nb1 or -Nb53 protein into Marc-145 cells or PAMs revealed a gradual increase first, followed by a gradual decrease; the intracellular TAT-Nb1 decreased by about half at 36 h. However, transduction was not observed in cells treated with the control Nb protein Nb1 (Figure 2A,B).

To further determine the entry and sub-cellular localization of transduced Nbs, TAT-Nb1 or -Nb53 were stained with anti-His mAb and a corresponding second antibody. As indicated in Figure 2E, the fluorescence intensity gradually increased with the increase in TAT-Nb1 or -Nb53 concentration. In addition, the fluorescence was mainly distributed in the cytoplasm, indicating that the transduced Nbs mainly existed in the cytoplasm after entering the target cells.

### 2.3. TAT-Nb1 Interacts with N Protein

**Figure 3:**
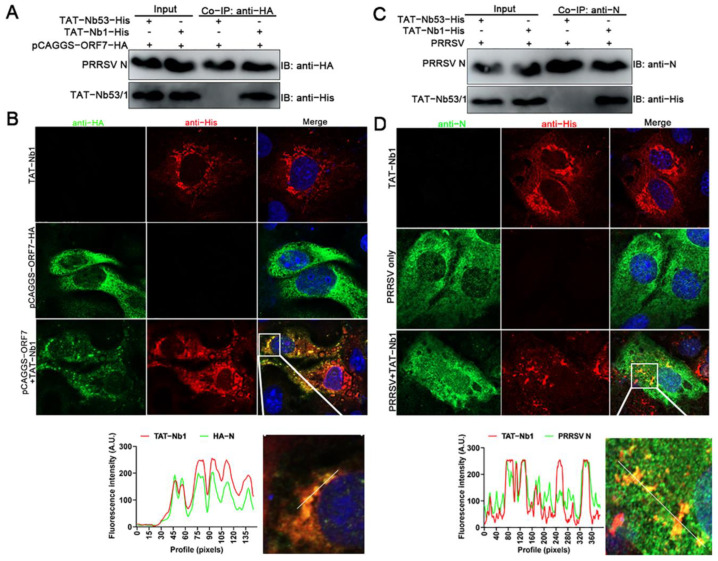
TAT-Nb1 interacts with PRRSV N Protein. Marc-145 cells seeded in 6-well plates were treated with 20 μM TAT-Nb1 for 6 h and then transfected with 1.5 μg of pCAGGS-ORF7-HA for another 24 h. Cells were harvested for co-IP analysis (**A**), and partial cells were fixed and analyzed using confocal (**B**). Marc-145 cells seeded in 6-well plates were incubated with 20 μM TAT-Nb1 for 6 h and followed by infection with 0.1 MOI of PRRSV for 24 h. Cells were collected for TAT-Nb1 and N protein interaction analysis using co-IP (**C**) and confocal (**D**), respectively. Scale bar = 20 μm.

To investigate the mechanism by which TAT-Nb exerts an anti-PRRSV effect, the interactions between the TAT-Nb1 or -Nb53 and PRRSV N protein were evaluated using co-immunoprecipitation (co-IP) and laser confocal assays. After TAT-Nb1 or TAT-Nb53 were expressed together with the PRRSV N protein, co-IP was performed using protein G Megbeads coated with anti-His mAb. As can be observed from Figure 3A, TAT-Nb1 interacted with the N protein expressed in HEK-293T cells, whereas TAT-Nb53 did not pull down the N protein. Co-localization analysis showed that the sub-cellular localization of TAT-Nb1 was almost identical with the eukaryotic-expressed N protein (Figure 3B).

Then, whether TAT-Nb1 or -Nb53 interacted with the N protein during PRRSV infection was also determined by co-IP. As indicated in Figure 3C, TAT-Nb1 (but not -Nb53) interacted with the PRRSV N protein during the viral infection of Marc-145 cells. Furthermore, the co-localization analysis showed that the sub-cellular localization of TAT-Nb1 was almost identical to the N protein during PRRSV infection of Marc-145 cells (Figure 3D). Taken together, these results suggest that the inhibitory effect of TAT-Nb1 on PRRSV infection and replication largely relies on its interaction with the viral N protein during PRRSV replication. The N protein plays an important role in PRRSV capsid formation, while the Nb–N dimer complex may disrupt the correct protein conformation of the N protein necessary for viral capsid formation, and thus abrogating viral replication.

### 2.4. TAT-Nb1 Suppresses PRRSV Replication in Marc-145 Cells

**Figure 4:**
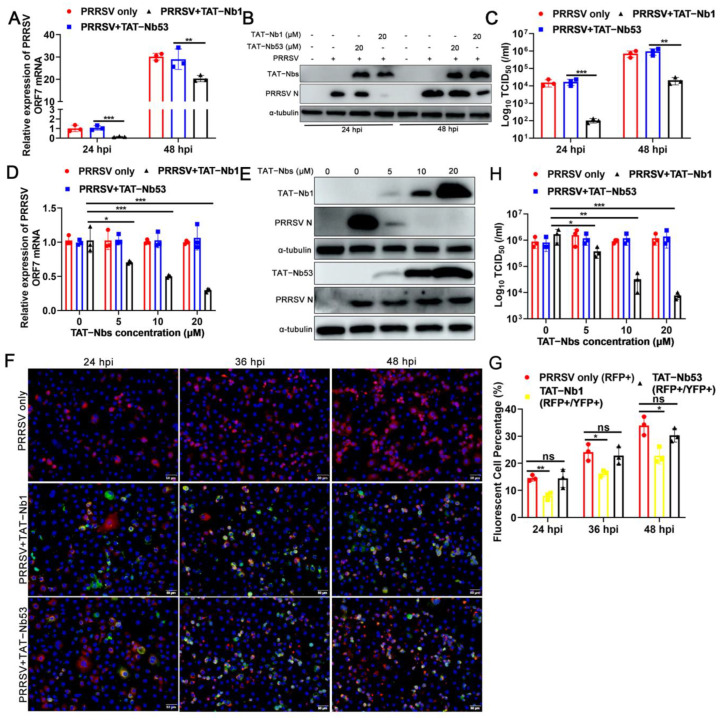
TAT-Nb1 inhibits PRRSV replication in Marc-145 cells. Marc-145 cells were seeded in 6-well plates (2 × 10^5^/well) and incubated overnight at 37 °C with 5% CO_2_ followed by treating with 20 μM TAT-Nb1 or -Nb53 for 6 h and infected with 0.1 MOI of PRRSV. Cells and supernatants were harvested at 24 and 48 hpi to detect the expression of PRRSV ORF7 mRNA and N protein using qPCR (**A**) and Western blot (**B**), respectively, and supernatant viral titers were analyzed using TCID_50_ (**C**). Marc-145 cells were treated with 0, 5, 10, and 20 μM TAT-Nb1 or -Nb53 for 6 h and then infected with PRRSV (0.1MOI). At 36 hpi, the expression of PRRSV ORF7 and N protein was analyzed using qPCR (**D**), Western blot (**E**), or IFA (**F**,**G**), respectively; supernatant viral titers were analyzed using TCID_50_ (**H**). α-Tubulin was detected simultaneously as an internal control to normalize the quantitative data. Data are expressed as the means ± standard deviations (SD) of the results of three independent experiments. *p* values were calculated using Student’s *t* test or analysis of variance (ANOVA). *, *p* < 0.05; **, *p* < 0.01; ***, *p* < 0.001; ns, not significant.

As PRRSV has a very restricted cell tropism, both in vivo and in vitro, Marc-145 cells were first chosen to assess the antiviral effect of TAT-Nb1 or -Nb53. Marc-145 cells treated with or without 20 μM TAT-Nb1 or -Nb53 were mock-infected or infected with 0.1 MOI of PRRSV. At 24 and 48 hpi, cells and supernatants were collected to determine the effects of TAT-Nb1 and -Nb53 on PRRSV replication. As revealed in Figure 4A,B, ORF7 mRNA and N protein expression levels were obviously decreased at both 24 and 48 hpi in the presence of TAT-Nb1, but not TAT-Nb53. Then, TCID_50_ was performed to detect progeny viral titers in supernatants. The TCID_50_ results revealed that the transduction of TAT-Nb1, but not -Nb53, obviously reduced supernatant viral titers at both 24 and 48 hpi (Figure 4C). To further authenticate the inhibitory effect of TAT-Nb1 on PRRSV infection, Marc-145 cells treated with different doses of TAT-Nb1 or -Nb53 were infected with PRRSV at an MOI of 0.1. The results suggested that TAT-Nb1 abrogated PRRSV ORF7 mRNA, as well as N protein expression, in a dose-dependent manner (Figure 4D,E); furthermore, TAT-Nb1 decreased supernatant progeny viral titers as well (Figure 4H). However, TAT-Nb53 exhibited no inhibitory effect on ORF7 mRNA, N protein expression, or supernatant progeny viral titers (Figure 4D,E,H). The IFA results also revealed that TAT-Nb1, but not TAT-Nb53, suppressed PRRSV N protein expression in a dose-dependent manner (Figure 4F,G). These results suggest that the transduction of TAT-Nb1 into Marc-145 cells effectively interrupted PRRSV replication.

### 2.5. TAT-Nb1 Inhibits PRRSV Replication in PAMs

**Figure 5:**
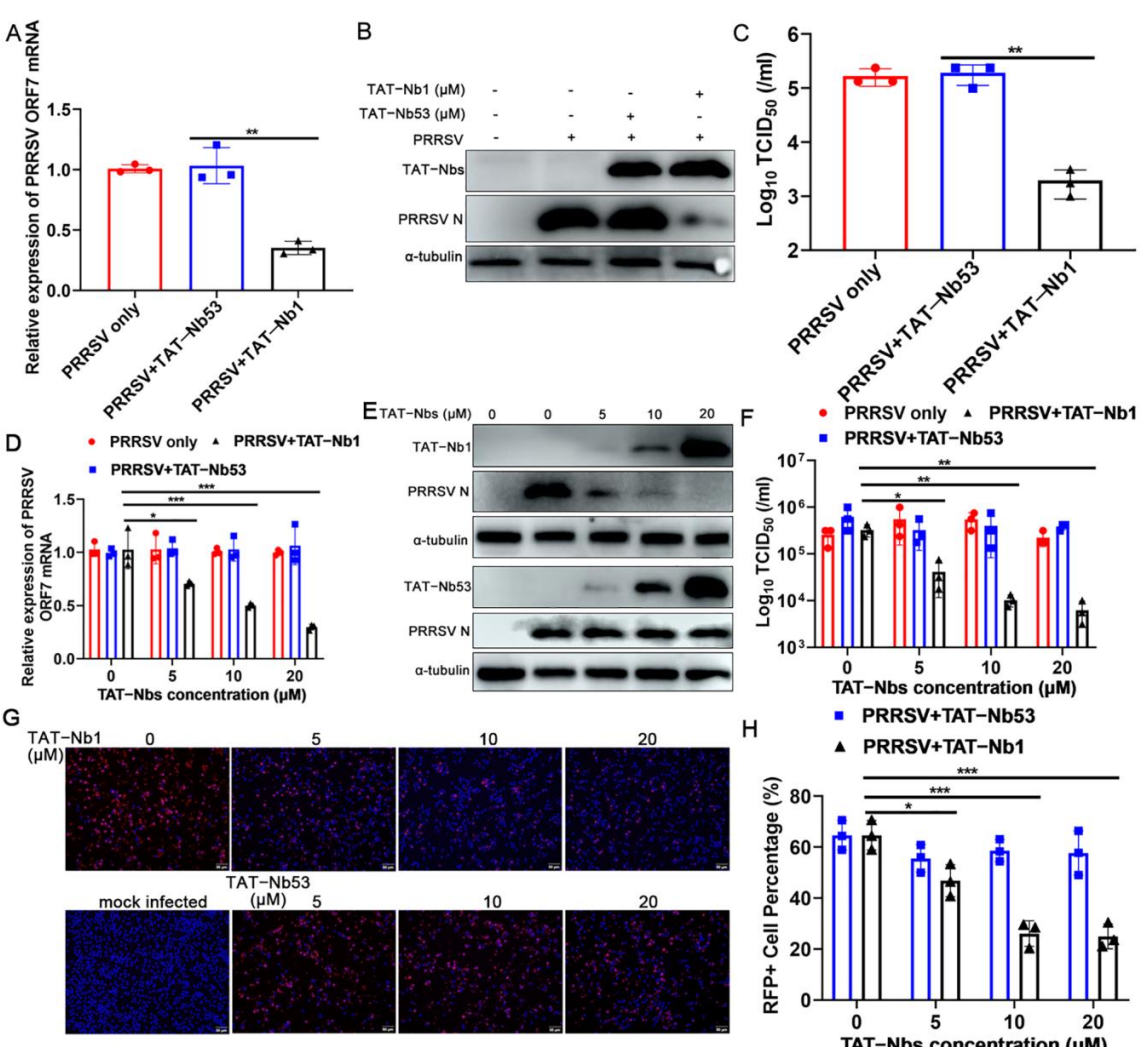
TAT-Nb1 inhibits PRRSV replication in PAMs. PAMs were seeded in 6-well plates (2 × 10^6^/well) and incubated overnight at 37 °C with 5% CO_2_ followed by treatment with 20 μM TAT-Nb1 or -Nb53 for 6 h and infected with 0.01 MOI of PRRSV. Cells and supernatants were harvested at 36 hpi to detect the expression of PRRSV ORF7 mRNA using qPCR (**A**), the expression of N protein using Western blot (**B**), and supernatant viral titers using TCID_50_ (**C**), respectively. PAMs were seeded in 6-well plates (0.5 × 10^6^/well) and incubated with 0, 5, 10, or 20 μM TAT-Nb1 or -Nb53 for 6 h and then infected with PRRSV (0.01 MOI). At 24 hpi, the expression of PRRSV ORF7 mRNA and N protein was determined by qPCR (**D**), Western blotting (**E**), and IFA (**G**,**H**), respectively. Supernatant progeny viral titers were determined by TCID_50_ (**F**). α-Tubulin was used as an internal control to normalize the quantitative data. Scale bar = 50 μm. Data are expressed as the means ± standard deviations (SD) of the results of three independent experiments. *p* values were calculated using Student’s *t* test or analysis of variance (ANOVA). *, *p* < 0.05; **, *p* < 0.01; ***, *p* < 0.001; ns, not significant.

Porcine alveolar macrophages (PAMs) are the main target cells for PRRSV replication and proliferation in vivo. Therefore, PAMs were also used to further validate the antiviral activity of TAT-Nb1. PAMs treated with or without 20 μM TAT-Nb1 or -Nb53 were mock-infected or infected with PRRSV at an MOI of 0.01. Cells and culture supernatants were harvested at 36 hpi, in order to evaluate the effect of TAT-Nb1 or -Nb53 on PRRSV replication. PRRSV ORF7 mRNA and N protein expression levels were obviously decreased at 36 hpi in the presence of TAT-Nb1, but not -Nb53 (Figure 5A,B). The TCID_50_ results indicated that the transduction of TAT-Nb1, but not TAT-Nb53, obviously reduced supernatant viral titers at 36 hpi (Figure 5C). To further certify the antiviral activity of TAT-Nb1, PAMs were treated with or not with 0, 5, 10, or 20 μM TAT-Nb1 or -Nb53 for 6 h, then infected with PRRSV (0.01 MOI). At 24 hpi, cells and culture supernatants were collected. PRRSV ORF7 mRNA and N protein expression levels were markedly decreased with the increase in TAT-Nb1 concentration (Figure 5D,E). The TCID_50_ results revealed that supernatant viral titers also decreased gradually, in a TAT-Nb1 concentration-dependent manner (Figure 5F). Likewise, no anti-PRRSV activity of TAT-Nb53 was observed (Figure 5D–F).

To further analyze the anti-PRRSV effect of TAT-Nb1, the expressions of viral N protein of either TAT-Nb1- or -Nb53-treated cells were analyzed by IFA. As shown in Figure 5G,H, the expression of N protein decreased in a TAT-Nb1 concentration-dependent manner. However, as the control, TAT-Nb53 did not exhibit a suppressive effect on PRRSV replication (Figure 5G,H). These results suggest that TAT-Nb1 suppresses PRRSV infection and replication in PAMs.

### 2.6. TAT-Nb1 Decreases PRRSV Replication without Inducing Cytotoxicity

**Figure 6:**
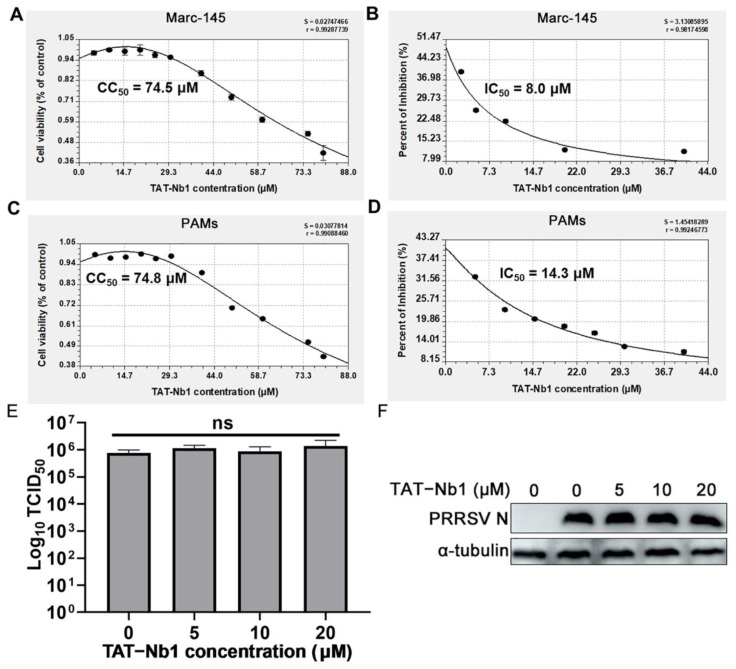
The CC_50_ and IC_50_ values of TAT-Nb1 in Marc-145 cells and PAMs. Marc-145 cells (**A**) or PAMs (**C**) were treated with various concentrations of TAT-Nb1 for 36 h and then subjected to CCK-8 assay. The viability curve and CC_50_ values were generated using CurveExpert software. Marc-145 cells (**B**) or PAMs (**D**) were treated with various concentrations of TAT-Nb1 for 6 h, then cells were infected with PRRSV (0.1 MOI) for 36 h. IFA was performed, and the inhibition curve and IC_50_ was generated using ImageJ and CurveExpert software. (**E**,**F**) PRRSV suspension (100 TCID_50_) was co-incubated with TAT-Nb1 at 37 °C for 1 h and inoculated to Marc-145 cells, and part of the supernatant was used to determine the virus titers directly. After 24 h, cells were harvested for viral replication analysis, and virus titer was tested using TCID_50_. Data are expressed as the means ± standard deviations (SD) of the results of three independent experiments. *p* values were calculated using analysis of variance (ANOVA). ns, not significant.

To determine whether the anti-PRRSV activity of TAT-Nb1 can be attributed to its cytotoxic effect, the CC_50_ and IC_50_ values of TAT-Nb1 in both Marc-145 cells and PAMs were determined. In Marc-145 cells, the CC_50_ and IC_50_ values of TAT-Nb1 were 74.5 μM and 8.0 μM, respectively, and the SI (CC_50_/IC_50_) was 9.31. In PAMs, the CC_50_ and IC_50_ values of TAT-Nb1 were 74.8 μM and 14.3 μM, respectively, and the SI was 5.23 (Figure 6A–D). TAT-Nb1 treatment did not inactivate PRRSV infectivity, as co-incubation of different doses of TAT-Nb1 with PRRSV particles did not inactivate PRRSV infectivity (Figure 6E) or viral infection of Marc-145 cells (Figure 6F). Collectively, these results suggest that TAT-Nb1 exerts antiviral activity without causing any cytotoxic effect.

### 2.7. TAT-Nb1 Inhibits the Replication of Multiple PRRSV Strains

**Figure 7:**
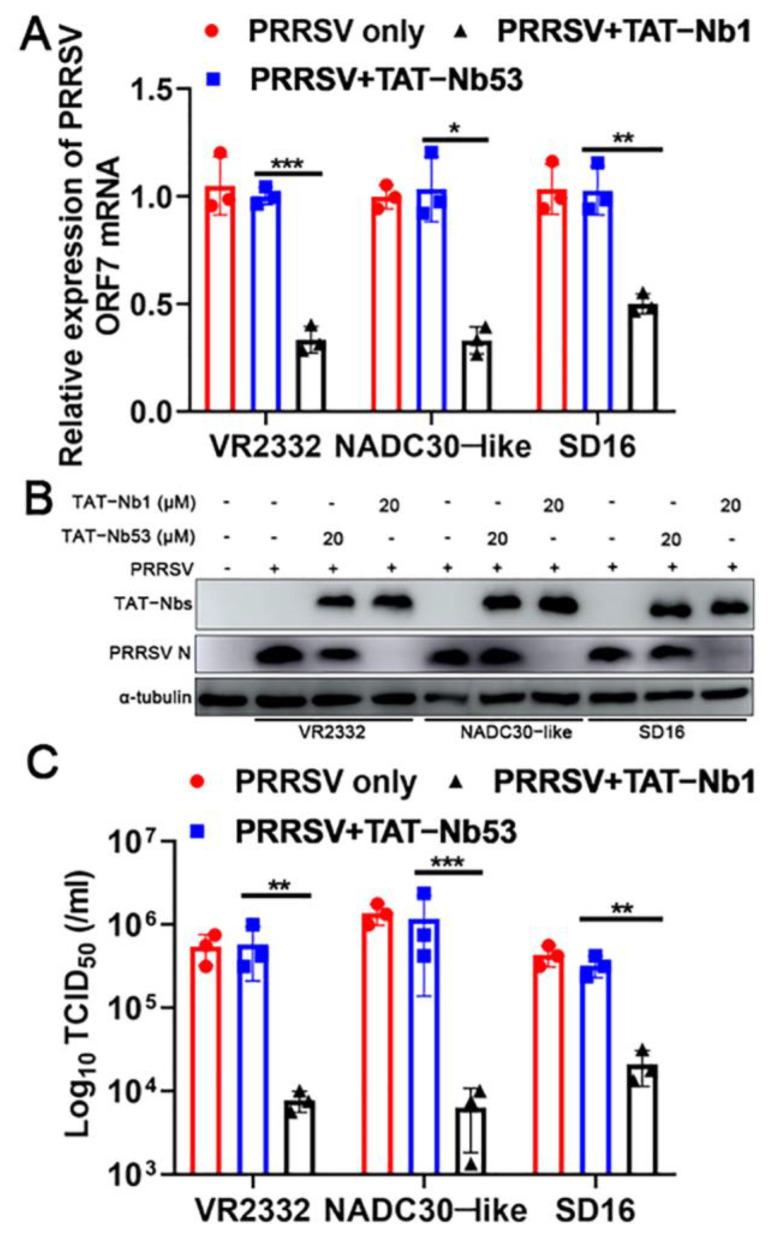
TAT-Nb1 inhibits PRRSV replication independently of viral strains. Marc-145 cells were seeded in 6-well plates at a density of 2 × 10^5^ cells/well. Then, 24 h later, cells were treated with 20 μM TAT-Nb1 or -Nb53 for 6 h and then infected with VR2332-, NADC30-like-, and SD16-PRRSV at an MOI of 0.1. Cells or supernatants were harvested at 24 hpi to determine the expression of PRRSV ORF7 and N protein using qPCR (**A**), Western blot (**B**), and the supernatant viral titers analysis using TCID_50_ (**C**), respectively. α-Tubulin was detected simultaneously as an internal control. Data are expressed as the means ± standard deviations (SD) of the results of three independent experiments. *p* values were calculated using Student’s *t* test. *, *p* < 0.05; **, *p* < 0.01; ***, *p* < 0.001.

To determine whether the antiviral effect of TAT-Nb1 was PRRSV strain-dependent, three PRRSV strains—including two HP-PRRSV strains (NADC30-like and SD16) and one LP-PRRSV type 2 strain (VR2332)—were inoculated to Marc-145 cells (MOI = 0.1) in the presence of 20 μM TAT-Nb1 or -Nb53, in order to further confirm the above conjecture. As shown in Figure 7A,B, TAT-Nb1 decreased ORF7 mRNA and N protein expression of different PRRSV strains, and virus titers were also simultaneously decreased (Figure 7C). Meanwhile, TAT-Nb53 did not display any anti-PRRSV effect (Figure 7A–C). Collectively, these data suggest that TAT-Nb1 can suppress the replication of multiple PRRSV strains.

## 3. Discussion

The PRRS epidemic has led to huge losses in the global pig industry [28]. Due to the high variability of the PRRSV genome, no commercial vaccines are able to provide complete protection. Furthermore, there are no effective drugs for treatment of PRRS. Considering the unique properties of nanobodies, they have been widely used in the development of therapeutic antibody drugs, diagnostic reagents, affinity purification substrates, and various other areas of scientific research [29,30]. Although high-affinity monoclonal antibodies have received priority attention in research and clinical applications as potential therapeutics, their production through mammalian cell expression is expensive [31,32]. In contrast, Nbs can be prepared using bacteria or yeast at an extremely low cost, which confers great advantages to Nbs as antiviral therapeutics. However, Nbs face several obstacles as therapeutic drugs, one of which is that they cannot directly cross biological barriers to target virus-associated proteins, due to the selective permeability of cell membranes.

The present results indicated that intracellular TAT-Nb1 decreased by about half at 36 h; however, TAT-Nb1 was still capable of suppressing PRRSV replication even at 48 hpi (Figure 4A-C), which may be attributed to the fact that TAT-Nb1 effectively inhibited viral replication in the early stage of PRRSV infection, where the antiviral effect lasted up to 48 hpi. This suggests that prolonging the half-life is an effective way to improve the antiviral effect of Nbs. Studies have shown that multivalent Nbs can better block viral replication [33,34]. However, in the present study, we only discussed the anti-PRRSV activity of monovalent Nbs; in future research, we intend to prepare multivalent Nbs and assess their blocking effect on PRRSV replication. To exclude non-specific results, two recombinant TAT-Nb proteins were constructed simultaneously, and we verified that TAT-Nb1 inhibited the replication of multiple PRRSV strains in Marc-145 cells and PAMs, providing a rationale for TAT-Nb1 as an antiviral agent (Figure 4, Figure 5, and Figure 7). In contrast, although TAT-Nb53 also entered the cell smoothly, it did not exert antiviral effects (Figure 4, Figure 5, and Figure 7). To analyze how TAT-Nb1 blocks the replication of PRRSV, the relationship between the N protein and Nb1 was analyzed. The results demonstrated that TAT-Nb1 interacts with the PRRSV N protein, whereas TAT-Nb53 does not (Figure 3), indicating that the binding of TAT-Nb1 and the N protein may affect the conformation of the N protein, which is necessary for the formation of the viral capsid. In fact, the PRRSV N protein undergoes dimerization in infected cells [35], and it is possible that the binding of Nbs to the N protein disrupts this dimerization. In addition, in the present study, we did not identify the interaction sites between TAT-Nb1 and the PRRSV N protein, which may be helpful in clarifying the molecular mechanism by which TAT-Nb1 blocks PRRSV replication. This aspect requires further in-depth study.

At present, the NADC30-like PRRSV strain is widely prevalent in pig farms in China and new mutant strains are constantly produced, which has caused ongoing harm to the pig industry. Our results show that, in addition to the classical VR2332 strain, TAT-Nb1 also effectively inhibited the infection by and replication of the NADC30-like PRRSV strain (Figure 7), indicating that it has broad-spectrum anti-PRRSV activity. As the most abundant viral protein in infected target cells, the PRRSV N protein is highly conserved among different type II strains but shows only about 80% homology between type I and type II [11,36,37]. The results of this study showed that TAT-Nb1 could effectively inhibit the replication of different type II strains, indicating that Nb1 is likely to target the conserved sites of the N protein; however, whether it has an inhibitory effect on type I PRRSV is still unclear, and further verification is required in this context.

While the in-depth mechanisms of how TAT-Nb1 blocks the replication of PRRSV are currently unknown, based on the available findings, one possible explanation is that TAT-Nb1 interacts with the specific domain of PRRSV N protein; however, the exact stage of PRRSV replication that TAT-Nb1 affects is still not known and, so, further studies are required. Crystal structure analysis provides a powerful tool for deep discussion of the structure and function of nanobody–protein complexes. In future research, we will focus on analyzing the crystal structure of TAT-Nb1–N protein complexes and determining their interaction sites to clarify the molecular mechanism by which TAT-Nb1 inhibits PRRSV replication.

## 4. Conclusions

The use of a cell-penetrating peptide successfully mediated the entry of specific Nbs into PRRSV-permissive cells. The PRRSV infection experiment results demonstrated that TAT-Nb1 suppressed PRRSV replication, and that the antiviral effect is independent of the viral strains considered. Furthermore, the antiviral effect of Nb1 was found to rely on its interaction with the viral N protein. Taken together, these results suggest that TAT-Nb1 suppresses PRRSV replication in vitro, and it may be developed as an effective anti-PRRSV drug.

## 5. Materials and Methods

### 5.1. Cells and Viruses

Marc-145 cells were cultured in Dulbecco’s modified Eagle’s medium (DMEM; Thermo Fisher Scientific, Inc.; Waltham, MA, USA) containing 10% fetal bovine serum (FBS, Gibco; Thermo Fisher Scientific, Inc.) and 1% penicillin–streptomycin (Thermo Fisher Scientific, Inc.). Cells were passaged approximately every 48 h. PAMs were isolated from lung tissue of 4–6-week-old specific-pathogen-free piglets and cultured in RPMI 1640 medium (Thermo Fisher Scientific, Inc.) containing 10% FBS (Gibco) and 1% penicillin–streptomycin (Thermo Fisher Scientific, Inc.). All cells were cultured in a humidified incubator at 37 °C with 5% CO_2_. The animal experiments in this study were approved by the Animal Protection and Use Committee of Henan Agricultural University (DWLL3086427.13), and strictly followed the official guidelines of the Animal Protection and Use Committee.

Most experiments were performed using a highly pathogenic (HP) PRRSV strain, GD-HD (GenBank ID: KP793736.1) [38], abbreviated as PRRSV unless otherwise specified. In addition, another two genotype 2 HP-PRRSV strains NADC30-like (a current epidemic strain in Chinese pig farms; GenBank ID: MH500776.1) [27] and SD16 (GenBank ID: JX087437), as well as one classical low pathogenic (LP) PRRSV strain VR2332 (GenBank ID: EF442771), were used for the viral inhibition experiments [38]. All of these viruses were propagated and titered on Marc-145 cells and stored at −80 °C until further use.

### 5.2. Antibodies and Reagents

Mouse monoclonal antibody (mAb) directly against His and rabbit polyclonal antibodies (pAb) directly against α-tubulin were purchased from Proteintech. Mouse mAb 6D10, which specifically reacts with the PRRSV N protein (1 μg/mL), has been described in our previous work [39]. HRP-conjugated goat anti-mouse and -rabbit IgG antibodies were obtained from Jackson ImmunoResearch Laboratories, Inc (West Grove, PA, USA). Alexa Fluor 594 conjugated goat anti-mouse IgG H&L and Alexa Fluor 488-conjugated goat anti-rabbit IgG H&L were both purchased from Abcam (Cambridge, UK).

### 5.3. Construction of Recombinant TAT-Nbs Proteins

The pET-21b plasmid with a His tag at the N-terminus was used to construct a recombinant plasmid expressing TAT-Nbs proteins. The sequences of Nb1 and Nb53 were as previously reported [27], and TAT-Nbs gene segments were obtained by overlap PCR. After digesting with *Nde* I and *Xho* I, the TAT-Nbs sequences were inserted into the pET-21b prokaryotic expression vector. The sequencing correct plasmids were then transformed into the expressing competent cell *E. coli* BL21 (DE3; TransGen Biotech, Beijing, China). Single colonies were selected, and the recombinant TAT-Nbs were induced with 1.0 mM of Isopropyl β-D-Thiogalactoside (IPTG) at 37 °C for 6 h. Bacteria were collected and sonicated in an ice bath, and the His-tag TAT-Nbs were purified with a Ni-NTA column. The concentrations of purified TAT-Nbs proteins were determined using a BCA kit, then aliquoted and stored at −80 °C until further use.

### 5.4. Western Blotting Analysis

After appropriate treatment, cell samples cultured in 6-well plates were harvested and lysed on ice using NP40 lysis buffer (Beyotime Biotechnology, Shanghai, China) containing phenylmethanesulfonyl fluoride (PMSF) and then centrifuged at 13,000 rpm and 4 °C for 10 min, in order to remove cell debris. Then, the clarified protein supernatants were boiled at 95 °C for 5 min in 1 × SDS loading buffer. The cellular proteins were then separated by 12.5% SDS-PAGE and transferred to PVDF membranes (Sigma-Aldrich, Merck KGaA, St. Louis, MO, USA). The membranes were first blocked with 5% skim milk in PBS’T at 4 °C overnight. Next, the membranes were probed with 6D10 (1:1000), mouse anti-His mAb (1:5000), and rabbit anti-α-tubulin pAb (1:5000), as well as corresponding second antibodies, to detect N protein, TAT-Nb1 or -Nb53, and α-tubulin, respectively. The target bands were visualized using enhanced chemiluminescence (ECL) reagent on an Amersham Imager 600 instrument (Cytiva, Washington, DC, USA).

### 5.5. Quantitative Real Time-PCR (qRT-PCR)

After the cells were treated accordingly, total RNA from mock-infected, PRRSV-infected Marc-145 cells, PAMs, or supernatants was extracted using TRizol Reagent following the manufacturer’s introductions (Thermo Fisher Scientific, lnc.). The concentration of total RNA was detected and 500 ng of total RNA of each sample was reverse transcribed into cDNA using a Primescript RT reagent Kit (TaKaRa, Dalian, China). Then, qRT-PCR was performed using the following primers using FastStart Universal SYBR Green Master (Roche, Basel, Switzerland) on an Applied Biosystems Quantity Studio system (Thermo Fisher Scientific, Inc.): ORF7, forward 5′-AGATCATCGCCCAACAAAAC-3′ and reverse 5′-GACACAATTGCCGCTCACTA-3′; β-actin, forward 5′-TCCCTGGA GAAGAGCTACGA-3′ and reverse 5′-AGCACTGTGTTGGCGTACAG-3′. The β-actin gene was detected simultaneously as an internal reference control for ORF7 expression normalization. The relative expression levels of ORF7 genes were quantified using the 2^−△△Ct^ method. 

### 5.6. TAT-Nbs Treatment and PRRSV Infection

Marc-145 cells (1.0 × 10^5^ cell/mL) or PAMs (0.5 × 10^6^ cell/mL) were seeded in 6-well plates for about 24 h before adding different concentrations (0, 5, 10, or 20 μM) of TAT-Nb1 or -Nb53 diluted with 3% FBS + DMEM and cultured for 6 h. After discarding the old culture medium, the cells were infected with 0.1 MOI of PRRSV and cultured for another 1 h at 37 °C with 5% CO_2_. The virus and DMEM mixture was discarded, cells were washed once using PBS, fresh 3% FBS + DMEM was added, and culturing was continued to the indicated time points. Whole cells were collected for the determination of PRRSV N protein, TAT-Nb1, or -Nb53 expression using Western blot. 

### 5.7. Viral Titration Assay

To determine the titers of progeny virus in cell culture supernatants, Marc-145 cells seeded in 96-well plates at a density of 1 × 10^4^ cells/well were cultured overnight at 37 °C with 5% CO_2_. The collected supernatant samples were diluted 10-fold continuously, and 100 μL was added to each well, with 8 duplicates for each sample, and incubated at 37 °C for 1 h. Then, the virus suspension was aspirated and supplemented with fresh 3% FBS + DMEM and continued to culture. Five to seven days after infection, the cytopathic effect (CPE) of each well was observed, and the Spearman–Karber method was used to calculate the 50% tissue culture infectious dose (TCID_50_).

### 5.8. Indirect Immunofluorescence Assay (IFA)

Marc-145 cells and PAMs growing on the glass slides treated with various doses of TAT-Nb1 or -Nb53 (0, 5, 10, and 20 μM) were mock-infected or infected with PRRSV (0.1 MOI). At indicated time points post viral infection, cells were fixed with 4% paraformaldehyde (500 μL/well) at room temperature (RT) for 10 min and then permeabilized with 0.3% Triton X-100 (200 μL/well) at RT for 3 min. An mAb 6D10 against PRRSV N protein at a 1:500 dilution or mouse anti-His mAb at a 1:1000 dilution was used as the primary antibody, and incubated with gentle shaking for 1 h at RT. The cells were then incubated with Alexa Fluor 594 conjugated goat anti-mouse IgG (H&L) or Alexa Fluor 488 conjugated goat anti-rabbit IgG (H&L) at a 1:300 dilution as the secondary antibody for 1 h in the dark at RT. Nuclei were stained with DAPI and slides were fixed using Fluoroshield (Sigma-Aldrich, Merck KGaA). All fluorescent images were acquired and analyzed using an inverted fluorescence microscope (Olympus Corporation, Tokyo, Japan).

### 5.9. Cell Viability and Cytotoxicity Assay

The cell viability and cytotoxicity associated with TAT-Nb1 and -Nb53 were evaluated using a Cell Counting Kit-8 (CCK-8; Beyotime Institute of Biotechnology, Shanghai, China). Briefly, Marc-145 cells (1 × 10^4^ cells/well) or PAMs (1 × 10^5^ cell/well) were seeded into 96-well cell culture plates and cultured in 10% FBS + DMEM at 37 °C in 5% CO_2_ for 24 h. The old culture medium was then replaced with new 3% FBS + DMEM containing 0, 5, 10, 15, 20, 25, 30, 40, 50, 60, 75, or 80 μM of TAT-Nb1 or -Nb53, then continued to culture for another 36 h. After aspirating and discarding the old medium, 10 μL of CCK-8 reagent was added to each well containing 100 μL of DMEM and incubated at 37 °C for 2 h. The absorbance of each well at 450 nm was detected using an epoch microplate spectrophotometer (BioTek Instruments, Inc., Winooski, VT, USA), and cell viability was measured based on the OD450 value. The 50% cytotoxic concentration (CC_50_, μmol/L) was calculated by comparing the cell viability of the TAT-Nb1 mock-treatment group with that of the TAT-Nb1-treatment group using the GraphPad Prism software (version 5.0; GraphPad Software, Inc., San Diego, CA, USA), and was defined as the concentration causing visible changes in 50% of intact cells. The maximum non-cytotoxic concentration (MNCC) was defined as the maximum working concentration of TAT-Nb1 or -Nb53 that did not display cytotoxic effects, as detected by CCK-8.

### 5.10. Cytopathic Effect Inhibition Assay

The suppressive effect of TAT-Nb1 on PRRSV replication was evaluated by CPE inhibition assay. Briefly, Marc-145 cells (1 × 10^4^ cell/well) or PAMs (1 × 10^5^ cell/well) seeded into 96-well cell culture plates were cultured, and when cells reached 80% confluence, cells were infected with PRRSV (MOI = 0.1) and treated with a 2-fold serial dilution of TAT-Nb1, where the MNCC was set as the highest concentration. After culturing at 37 °C in 5% CO_2_ for 48 h, the CPE of each well was determined under a light microscope. Marc-145 cells or PAMs infected with PRRSV without TAT-Nb1 treatment were used as the control group. The TAT-Nb1 doses leading to 50% CPE decrease of target cells, when compared with the control group, were calculated and determined as the half-maximal inhibitory concentration (IC_50_, μmol/L). The ratio of CC_50_ to IC_50_ was calculated and determined as the selectivity index (SI).

### 5.11. Co-Immunoprecipitation Assay

HEK-293T cells were inoculated with 20 μM of TAT-Nb1 or -Nb53 for 6 h, followed by transfection with 2.0 μg pCAGGS-ORF7-HA eukaryotic expression plasmids. After transfection for 48 h, cell samples were harvested and lysed on ice using NP40 lysis buffer containing PMSF. To prepare bead–antibody–antigen complexes, 2 μg of the indicated antibodies were first bound to 40 μL Protein G Megbeads overnight at 4 °C, followed by washing 3 times with PBS. Then, the cell lysates were supplemented, with rotating and incubating for 4 h at 4 °C. After washing 3 times with PBS, the beads attached or unattached to target proteins were boiled with 2 × SDS loading buffer and analyzed by Western blotting. To further confirm the interaction between TAT-Nb1 or -Nb53 and N protein during PRRSV infection, Marc-145 cells incubated with TAT-Nb1 or -Nb53 for 6 h were mock-infected or infected with PRRSV at an MOI of 0.1. At 48 h post-infection (hpi), cell samples were collected, lysed, and subjected to co-IP analysis.

### 5.12. Statistical Analysis

All experiments above were performed independently at least three times, in order to ensure that the results were reproducible. Statistical significance was determined by Student’s *t*-test when two groups were compared, or by one-way analysis of variance (ANOVA) when more than two groups were compared. A *p*-value of <0.05 was considered to be statistically significant.

## Data Availability

The data sets and/or analyzed during the current study are available from the corresponding author on reasonable request.

## References

[1] Hanada K., Suzuki Y., Nakane T., Hirose O., Gojobori T. (2005). The origin and evolution of porcine reproductive and respiratory syndrome viruses. Mol. Biol. Evol..

[2] Yan X., Shang P., Yim-Im W., Sun Y., Zhang J., Firth A., Lowe J., Fang Y. (2022). Molecular characterization of emerging variants of PRRSV in the United States: New features of the -2/-1 programmed ribosomal frameshifting signal in the nsp2 region. Virology.

[3] Wei C., Dai A., Fan J., Li Y., Chen A., Zhou X., Luo M., Yang X., Liu J. (2019). Efficacy of Type 2 PRRSV vaccine against challenge with the Chinese lineage 1 (NADC30-like) PRRSVs in pigs. Sci. Rep..

[4] Bai X., Wang Y., Xu X., Sun Z., Xiao Y., Ji G., Li Y., Tan F., Li X., Tian K. (2016). Commercial vaccines provide limited protection to NADC30-like PRRSV infection. Vaccine.

[5] Snijder E.J., Kikkert M., Fang Y. (2013). Arterivirus molecular biology and pathogenesis. J. Gen. Virol..

[6] Meulenberg J.J. (2000). PRRSV, the virus. Vet. Res..

[7] Wensvoort G., Terpstra C., Pol J., Laak E., Bloemraad M., de Kluyver E., Kragten C., van Buiten L., den Besten A., Wagenaar F. (1991). Mystery swine disease in The Netherlands: The isolation of Lelystad virus. Vet. Q..

[8] Benfield D.A., Nelson E., Collins J., Harris L., Goyal S., Robison D., Christianson W., Morrison R., Gorcyca D., Chladek D. (1992). Characterization of swine infertility and respiratory syndrome (SIRS) virus (isolate ATCC VR-2332). J. Vet. Diagn. Investig..

[9] Meng X.J., Paul P., Halbur P., Lum M. (1995). Phylogenetic analyses of the putative M (ORF 6) and N (ORF 7) genes of porcine reproductive and respiratory syndrome virus (PRRSV): Implication for the existence of two genotypes of PRRSV in the U.S.A. and Europe. Arch. Virol..

[10] Lunney J., Fang Y., Ladinig A., Chen N., Li Y., Rowland B., Renukaradhya G. (2016). Porcine Reproductive and Respiratory Syndrome Virus (PRRSV): Pathogenesis and Interaction with the Immune System. Annu. Rev. Anim. Biosci..

[11] Doan D.N., Dokland T. (2003). Structure of the nucleocapsid protein of porcine reproductive and respiratory syndrome virus. Structure.

[12] Rossow K.D., Collins J., Goyal S., Nelson E., Christopher-Hennings J., Benfield D. (1995). Pathogenesis of porcine reproductive and respiratory syndrome virus infection in gnotobiotic pigs. Vet. Pathol..

[13] Kim H.S., Kwang J., Yoon I., Joo H., Frey M. (1993). Enhanced replication of porcine reproductive and respiratory syndrome (PRRS) virus in a homogeneous subpopulation of MA-104 cell line. Arch. Virol..

[14] Li C., Tang Z., Hu Z., Wang Y., Yang X., Mo F., Lu X. (2018). Natural Single-Domain Antibody-Nanobody: A Novel Concept in the Antibody Field. J. Biomed. Nanotechnol..

[15] Mir M.A., Mehraj U., Sheikh B., Hamdani S. (2020). Nanobodies: The "Magic Bullets" in therapeutics, drug delivery and diagnostics. Hum. Antibodies.

[16] De Genst E., Silence K., Decanniere K., Conrath K., Loris R., Kinne J., Muyldermans S., Wyns L. (2006). Molecular basis for the preferential cleft recognition by dromedary heavy-chain antibodies. Proc. Natl. Acad. Sci. USA.

[17] Hong J., Kwon H., Cachau R., Chen C., Butay K., Duan Z., Li D., Ren H., Liang T., Zhu J. (2022). Dromedary camel nanobodies broadly neutralize SARS-CoV-2 variants. Proc. Natl. Acad. Sci. USA.

[18] Wang L., Zhang L., Huang B., Li K., Hou G., Zhao Q., Wu C., Nan Y., Du T., Mu Y. (2019). A Nanobody Targeting Viral Nonstructural Protein 9 Inhibits Porcine Reproductive and Respiratory Syndrome Virus Replication. J. Virol..

[19] Koren E., Torchilin V.P. (2012). Cell-penetrating peptides: Breaking through to the other side. Trends Mol. Med..

[20] Vale N., Duarte D., Silva S., Correia A., Costa B., Gouveia M., Ferreira A. (2020). Cell-penetrating peptides in oncologic pharmacotherapy: A review. Pharmacol. Res..

[21] Sadeghian I., Heidari R., Sadeghian S., Raee M., Negahdaripour M. (2022). Potential of cell-penetrating peptides (CPPs) in delivery of antiviral therapeutics and vaccines. Eur. J. Pharm. Sci. Off. J. Eur. Fed. Pharm. Sci..

[22] Ramsey J.D., Flynn N.H. (2015). Cell-penetrating peptides transport therapeutics into cells. Pharmacol. Ther..

[23] Brooks H., Lebleu B., Vives E. (2005). Tat peptide-mediated cellular delivery: Back to basics. Adv. Drug Deliv. Rev..

[24] Torchilin V.P. (2008). Tat peptide-mediated intracellular delivery of pharmaceutical nanocarriers. Adv. Drug Deliv. Rev..

[25] Van Audenhove I., Gettemans J. (2016). Nanobodies as Versatile Tools to Understand, Diagnose, Visualize and Treat Cancer. EBioMedicine.

[26] Zhang N., Guo H., Zheng W., Wang T., Ma X. (2016). Design and screening of a chimeric survivin-specific nanobody and its anticancer activities in vitro. Anticancer Drugs.

[27] Duan H., Chen X., Zhao J., Zhu J., Zhang G., Fan M., Zhang B., Wang X., Sun Y., Liu B. (2021). Development of a Nanobody-Based Competitive Enzyme-Linked Immunosorbent Assay for Efficiently and Specifically Detecting Antibodies against Genotype 2 Porcine Reproductive and Respiratory Syndrome Viruses. J. Clin. Microbiol..

[28] Guo Z., Chen X., Li R., Qiao S., Zhang G. (2018). The prevalent status and genetic diversity of porcine reproductive and respiratory syndrome virus in China: A molecular epidemiological perspective. Virol. J..

[29] Wang Y., Fan Z., Shao L., Kong X., Hou X., Tian D., Sun Y., Xiao Y., Yu L. (2016). Nanobody-derived nanobiotechnology tool kits for diverse biomedical and biotechnology applications. Int. J. Nanomed..

[30] Zhu S., Luo F., Zhu B., Ling F., Wang E., Liu T., Wang G. (2021). A Nanobody-Mediated Virus-Targeting Drug Delivery Platform for the Central Nervous System Viral Disease Therapy. Microbiol. Spectr..

[31] Baum A., Fulton B., Wloga E., Copin R., Pascal K., Russo V., Giordano S., Lanza K., Negron N., Ni M. (2020). Antibody cocktail to SARS-CoV-2 spike protein prevents rapid mutational escape seen with individual antibodies. Science.

[32] Zost S.J., Gilchuk P., Case J., Binshtein E., Chen R., Nkolola J., Schafer A., Reidy J., Trivette A., Nargi R. (2020). Potently neutralizing and protective human antibodies against SARS-CoV-2. Nature.

[33] Lu Q., Zhang Z., Li H., Zhong K., Zhao Q., Wang Z., Wu Z., Yang D., Sun S., Yang N. (2021). Development of multivalent nanobodies blocking SARS-CoV-2 infection by targeting RBD of spike protein. J. Nanobiotechnol..

[34] Koenig P.A., Das H., Liu H., Kummerer B., Gohr F., Jenster L., Schiffelers L., Tesfamariam Y., Uchima M., Wuerth J. (2021). Structure-guided multivalent nanobodies block SARS-CoV-2 infection and suppress mutational escape. Science.

[35] Dokland T. (2010). The structural biology of PRRSV. Virus Res..

[36] Dea S., Gagnon C., Mardassi H., Pirzadeh B., Rogan D. (2000). Current knowledge on the structural proteins of porcine reproductive and respiratory syndrome (PRRS) virus: Comparison of the North American and European isolates. Arch. Virol..

[37] Meulenberg J.J., Bende R., Pol J., Wensvoort G., Moormann R. (1995). Nucleocapsid protein N of Lelystad virus: Expression by recombinant baculovirus, immunological properties, and suitability for detection of serum antibodies. Clin. Diagn. Lab. Immunol..

[38] Zhang A., Duan H., Li N., Zhao L., Pu F., Huang B., Wu C., Nan Y., Du T., Mu Y. (2017). Heme oxygenase-1 metabolite biliverdin, not iron, inhibits porcine reproductive and respiratory syndrome virus replication. Free Radic. Biol. Med..

[39] Zhang A.K., Duan H., Zhao H., Liao H., Du Y., Li L., Jiang D., Wan B., Wu Y., Ji P. (2020). Interferon-Induced Transmembrane Protein 3 Is a Virus- Associated Protein Which Suppresses Porcine Reproductive and Respiratory Syndrome Virus Replication by Blocking Viral Membrane Fusion. J. Virol..

